# Crystal structures of two Cu^II^ compounds: *catena*-poly[[chlorido­copper(II)]-μ-*N*-[eth­oxy(pyridin-2-yl)methyl­idene]-*N*′-[oxido(pyridin-3-yl)methyl­idene]hydrazine-κ^4^ *N*,*N*′,*O*:*N*′′] and di-μ-chlorido-1:4κ^2^ *Cl*:*Cl*-2:3κ^2^ *Cl*:*Cl*-di­chlorido-2κ*Cl*,4κ*Cl*-bis[μ_3_-eth­oxy(pyridin-2-yl)methano­lato-1:2:3κ^3^ *O*:*N*,*O*:*O*;1:3:4κ^3^ *O*:*O*:*N*,*O*]bis­[μ_2_-eth­oxy(pyridin-2-yl)methano­lato-1:2κ^3^ *N*,*O*:*O*;3:4κ^3^ *N*,*O*:*O*]tetracopper(II)

**DOI:** 10.1107/S2056989019008922

**Published:** 2019-06-28

**Authors:** Ousmane Sall, Farba Bouyagui Tamboura, Adama Sy, Aliou Hamady Barry, Elhadj Ibrahima Thiam, Mohamed Gaye, Javier Ellena

**Affiliations:** a Département de Chimie, Faculté des Sciences et Techniques, Université Cheikh Anta Diop, Dakar, Senegal; b Département de Chimie, Faculté des Sciences et Techniques, Université Alioune Diop, Bambey, Senegal; c Département de Chimie, Faculté des Sciences et Techniques, Université de Nouakchott, Nouakchott, Mauritania; d Instituto de Física de São Carlos, Universidade de São Paulo, CP 369, 13.560-970 – São Carlos, SP, Brazil

**Keywords:** crystal structure, copper complex, coordination polymer, open-cube structure

## Abstract

Two Cu^II^ complexes [Cu(C_14_H_13_N_4_O_2_)Cl]_*n*_, **I**, and [Cu_4_(C_8_H_10_NO_2_)_4_Cl_4_]_*n*_, **II**, have been synthesized. In the structure of the mononuclear complex **I**, each ligand is coordinated to two metal centers. The basal plane around the Cu^II^ cation is formed by one chloride anion, one oxygen atom, one imino and one pyridine nitro­gen atom. The apical position of the distorted square-pyramidal geometry is occupied by a pyridine nitro­gen atom from a neighbouring unit, leading to infinite one-dimensional polymeric chains along the *b-*axis direction. Each chain is connected to adjacent chains by inter­molecular C—H⋯O and C—H⋯Cl inter­actions, leading to a three-dimensional network structure. The tetra­nuclear complex **II** lies about a crystallographic inversion centre and has one core in which two Cu^II^ metal centers are mutually inter­connected *via* two enolato oxygen atoms while the other two Cu^II^ cations are linked by a chloride anion and an enolato oxygen. An open-cube structure is generated in which the two open-cube units, with seven vertices each, share a side composed of two Cu^II^ ions bridged by two enolato oxygen atoms acting in a μ_3_-mode. The Cu^II^ atoms in each of the two CuO_3_NCl units are connected by one μ_2_-O and two μ_3_-O atoms from deprotonated hydroxyl groups and one chloride anion to the three other Cu^II^ centres. Each of the penta­coordinated Cu^II^ cations has a distorted NO_3_Cl square-pyramidal environment. The Cu^II^ atoms in each of the two CuO_2_NCl_2_ units are connected by μ_2_-O and μ_3_-O atoms from deprotonated alcohol hy­droxy groups and one chloride anion to two other Cu^II^ ions. Each of the penta­coordinated Cu^II^ cations has a distorted NO_2_Cl_2_ square-pyramidal environment. In the crystal, a series of intra­molecular C—H⋯O and C—H⋯Cl hydrogen bonds are observed in each tetra­nuclear monomeric unit, which is connected to four tetra­nuclear monomeric units by inter­molecular C—H⋯O hydrogen bonds, thus forming a planar two-dimensional structure in the (01) plane.

## Abstract 2

A linear polymeric Cu^II^ complex and a Cu^II^ open-cube complex were synthesized using the tridentate Schiff base ligand, {1-[1-(pyrid­yl)(2-eth­oxy­ethyl­idene)]-2-[(pyridin-3-yl)carbon­yl]}hydrazine and copper(II) acetate and their mol­ecular and crystal structures determined.

## Chemical context   

Picolinic acid esters (González-Duarte *et al.*, 1996 ▸, 1998 ▸; Hay & Clark, 1979 ▸; Luo *et al.*, 2002 ▸; Paul *et al.*, 1974 ▸) as well as nicotinic acid hydrazide (Bharati *et al.*, 2015 ▸; Galić *et al.*, 2011 ▸; Nakanishi & Sato, 2017 ▸) are widely used in coordination chemistry for their ability to bind metals through the amino and/or the ester functional groups (Hay & Clark, 1979 ▸). Complexes formed by ethyl picolinate (EP) with various divalent metal thio­cyanates (Paul *et al.*, 1975 ▸), chlorides (González-Duarte *et al.*, 1996 ▸) and perchlorates (Natun *et al.*, 1995 ▸) have been prepared and characterized. Several modes of coordination are observed, depending on the conformation of the mol­ecule. Ethyl picolinate acts as a bidentate ligand coordinating through the ring nitro­gen and the carbonyl oxygen. The carb­oxy­lic ester function can coordinate in several ways, while the pyridine nitro­gen atom can also coordinate in a unidentate fashion. The nicotinic acid hydrazide can coordinate through the hydrazino moiety as well as through the pyridine nitro­gen atom (Lumme *et al.*, 1984 ▸; Shahverdizadeh *et al.*, 2011*a* ▸,*b* ▸). These facts make these ligands and their analogues very attractive and they have been used in several studies. Many polynuclear complexes of transition metals with various structures can be generated, depending on the disposition of the metal ions and the donor sites (N or O). Trimers (Zhang *et al.*, 2009 ▸), square shapes (Aouaidjia *et al.*, 2017 ▸), cyclic forms (Acevedo-Chávez *et al.*, 2002 ▸) and cubans (Shit *et al.*, 2013 ▸) have been reported that have potential applications in the field of magnetism (Shit *et al.*, 2013 ▸), catalysis (Okeke *et al.*, 2018 ▸) and biomimetic synthesis (Wu *et al.*, 2004 ▸). By extension, the introduction of an eth­oxy-carbonyl group in the *ortho* position of the pyridine gives a ligand that can have a similar behavior to α-amino acid esters. It has been shown that the presence of metal ions promotes the hydrolysis of the ester function of the picolinic ester (Xue *et al.*, 2016 ▸). A condensation can then occur between nicotinic acid hydrazide and the hydrolysed picolinic ester, to generate two organic ligands with a large number of coordination sites *in situ*, in the presence of copper(II) ions. These ligands then coordinate to the copper(II) cations to yield the two complexes that are reported here.
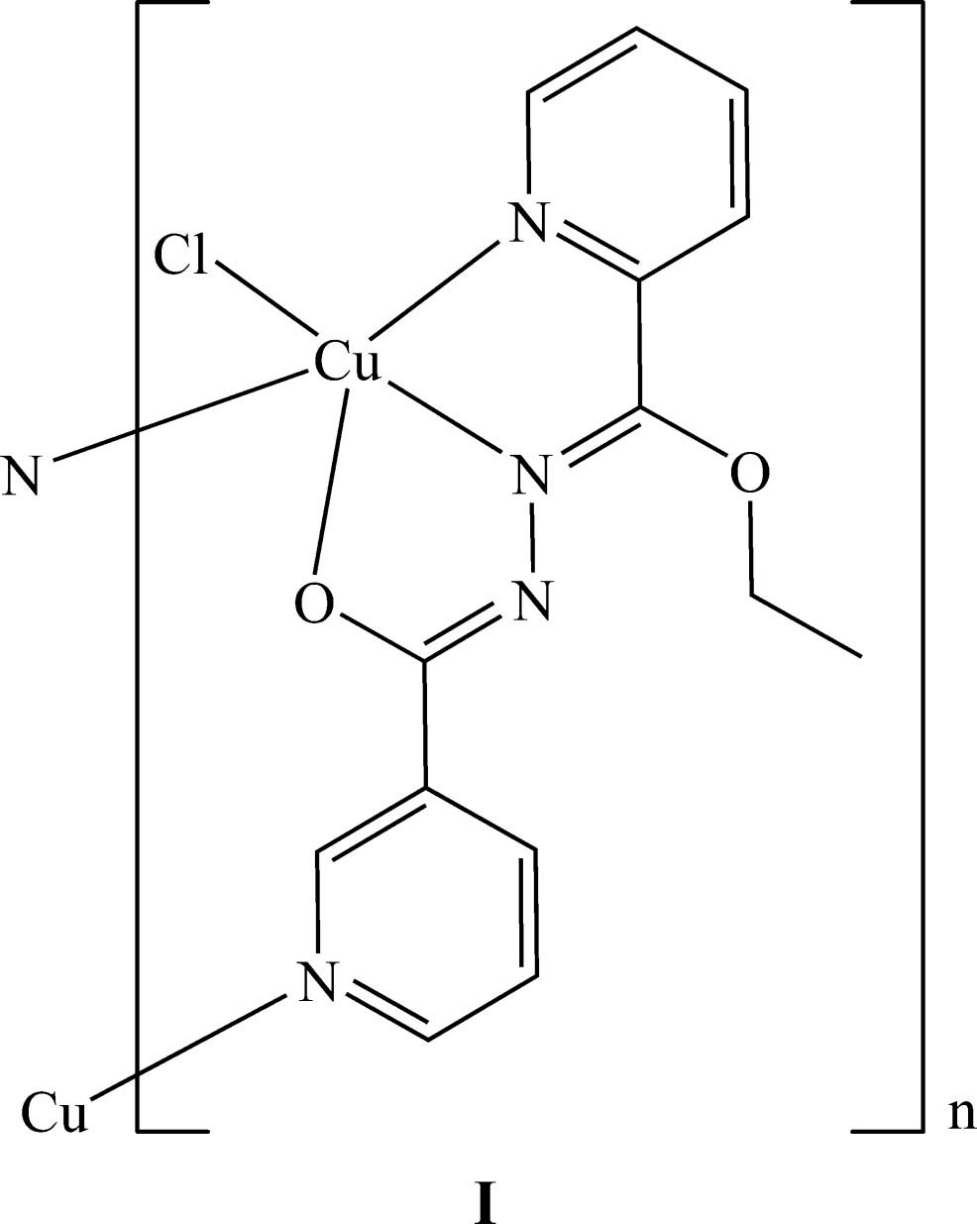
 
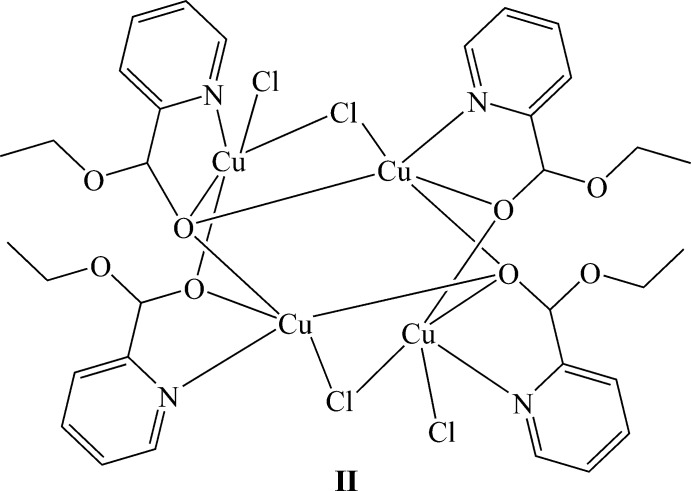


## Structural commentary   

The condensation reaction of pyridine-2-carbaldehyde and nicotinic acid hydrazide in ethanol in the presence of copper acetate yields two different complexes whose ligands are respectively a hemiacetal [eth­oxy(pyridine-2-yl)methanol] and a condensation product [({1-[1-eth­oxy-1-(pyridin-2-yl)methyl­ene]}-2-(oxonicotin­yl))hydrazine]. It has been shown (Papaefsta­thiou *et al.*, 2000 ▸; Boudalis *et al.*, 2008 ▸; Mautner *et al.*, 2010 ▸) that the presence of a metal can induce a nucleophilic attack of the ethanol mol­ecule on the carbonyl group to give a hemiacetal. This reaction can also occur when a fragment such as a pyridyl nitro­gen atom is present that is capable of inducing the polarization of the carbonyl function (Papaefsta­thiou *et al.*, 2000 ▸). It is under these conditions that the complexes **I** and **II** were formed *in situ*.

**Figure 1:**
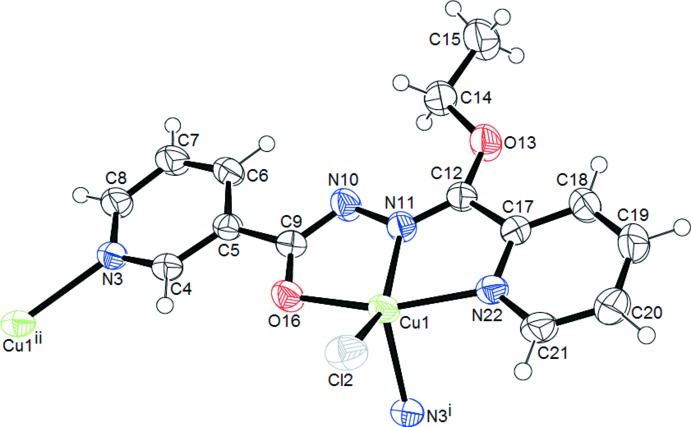
An *ORTEP* view of the repeat unit of the coordination polymer **I**, showing the atom-numbering scheme. Displacement ellipsoids are drawn at the 50% probability level. Symmetry codes: (i) 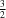 − *x*, −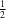 + *y*, 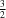 − *z*; (ii) 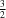 − *x*, 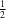 + *y*, 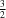 − *z*.

**Figure 2:**
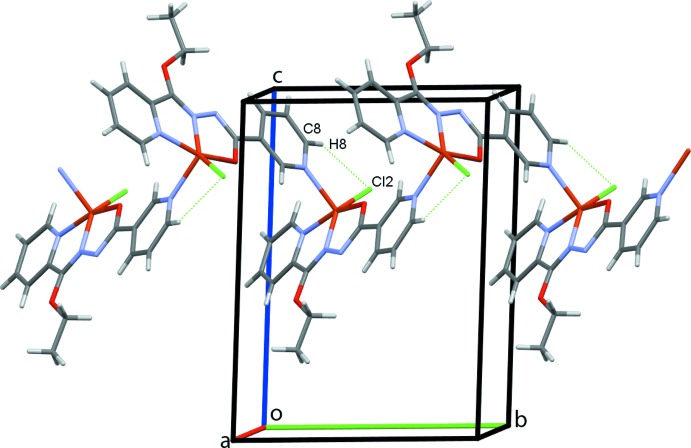
The polymer expansion of complex **I**, showing an infinite chain propagating along the *b*-axis direction. In this and subsequent figures, hydrogen bonds are drawn as dashed lines.

**Figure 3:**
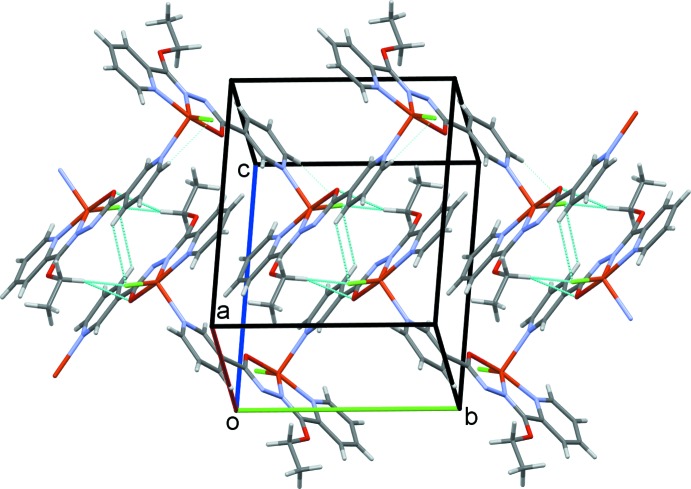
A view of the crystal packing of complex **I**.

In the crystal structure of the coordination polymer [CuCl(C_14_H_13_N_4_O_2_)]_*n*_, **I**, the repeat unit of which is shown in (Fig. 1 ▸), the Cu^II^ center is penta­coordinated by one chloride atom, one enolate oxygen atom of the mono deprotonated organic ligand, one pyridine, one imino nitro­gen atom, and by a pyridine nitro­gen atom of a ligand from an adjacent complex mol­ecule. This latter contact bridges the Cu^II^ cations to form a one-dimensional coordination polymer along the *b-*axis direction (Fig. 2 ▸). Inter­molecular C—H⋯O and C—H⋯Cl hydrogen bonds, (Table 1 ▸), link the polymers into a three-dimensional network (Fig. 3 ▸). The coordination environment can be best described as strongly distorted square pyramidal. The basal plane around the Cu^II^ ion is formed by the Cl2 anion with a Cu1—Cl2 distance of 2.2707 (6) Å, an O16 atom with a Cu1—O16 distance of 1.9808 (15) Å and the N11 and N22 atoms from the same ligand with a Cu—N distances of 1.9437 (17) and 2.0444 (17) Å (Table 2 ▸). These bond lengths are similar to the values found in related complexes (Datta *et al.*, 2011*a* ▸,*b* ▸; Da Silva *et al.*, 2013 ▸). The apical position of the distorted square pyramid is occupied by one pyridine N3 atom of a neighbouring unit with a Cu—N distance of 2.2009 (17) Å. This distance is shorter than that found in similar compound (Roztocki *et al.*, 2015 ▸). The ligand, which acts in a tridentate fashion, forms two five-membered rings upon coordination with the Cu^II^ centre: OCNNCu and NCCNCu, with the N11 atom common to both. The five-membered chelate rings impose large distortions on the ideal angles of a regular square pyramid, with bite angles in the range 79.11 (7)–79.40 (7)°, which are slightly smaller than those found in similar compounds (Roztocki *et al.*, 2015 ▸). The *transoid* angles in the basal plane O16—Cu1—N22 and N11—Cu1—Cl2 deviate severely from linearity with values of 158.51 (7)° and 146.17 (6)° (Table 2 ▸). These two largest angles around the Cu^II^ ion give a *τ* parameter of 0.206, which is indicative of a distorted square-pyramidal environment around the Cu^II^ ion (Addison *et al.*, 1984 ▸).

**Figure 4:**
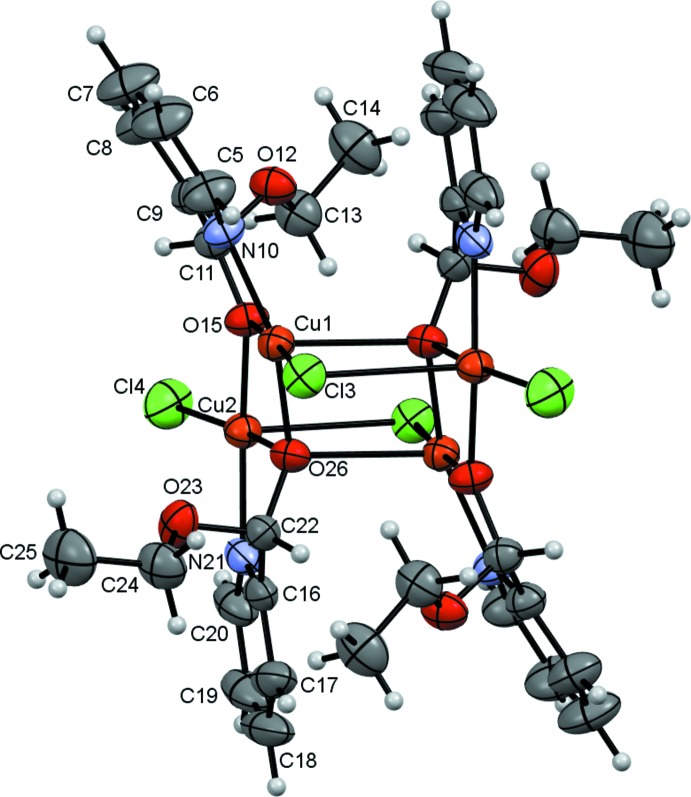
The structure of **II** with ellipsoids drawn at the 50% probability level. Unlabelled atoms are generated by the symmetry operation 1 − *x*, 1 − *y*, 1 − *z*.

**Figure 5:**
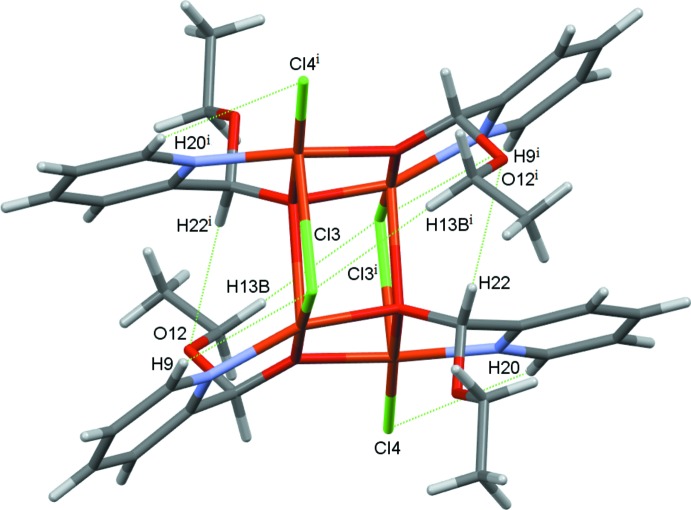
Intra­molecular hydrogen bonds in the structure of **II**. Symmetry code: (i) 1 − *x*, 1 − *y*, 1 − *z*.

In **II**, the tetra­nuclear open-cube complex lies about a crystallographic inversion centre, with each mono deprotonated eth­oxy(pyridin-2-yl)methano­late ligand coordinating to each Cu atom through its imine nitro­gen atom and its alcoholate oxygen atom, forming five-membered chelate rings (Fig. 4 ▸). The mol­ecule also forms intra­molecular hydrogen bonds between a terminal chloride atom and an aromatic hydrogen atom (C20—H20⋯Cl4) and between a bridging chloride and both an aromatic and a methyl­ene hydrogen atom (C9—H9⋯Cl3 and C13—H13*B*⋯Cl3^i^). Intra­molecular C—H⋯O contacts are also found (Table 3 ▸, Fig. 5 ▸). There are two discrete Cu^II^ environments, Cu1NO_3_Cl and Cu2NO_2_Cl_2_. Two mol­ecules of the ligand act as bridges between two neighbouring Cu atoms through their alcoholate atoms in a μ_2_ mode while the other two ligand mol­ecules bridge in a μ_3_ fashion. The structure consists of two Cu_3_O_3_Cl cores. The first core comprises Cu1, Cu1^i^, Cu2 atoms μ_3_-bridging atoms O26, O26^i^, a μ_2_-bridging O15 atom and a μ_2_-bridging Cl3^i^ ion [symmetry code: (i) −*x* + 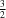, *y* − 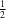, −*z* + 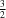)]. The second comprises Cu1, Cu1^i^, Cu2^i^ atoms, μ_3_-bridging atoms O26, O26^i^, a μ_2_-bridging O15^i^ atom and a μ_2_-bridging Cl3 ion. The result is a is a distorted open-cube, defined as a distorted cube missing one corner. This can be seen by considering that the range of Cu—O—Cu angles is [99.76 (6)–102.98 (6)°] and the Cu1—Cl3—Cu2^i^ angle is 84.39 (2)°. These differ extensively from the 90° angles of an ideal cube. The two Cu_3_O_3_Cl open-cubes are joined by a perfectly rectangular side defined by the Cu1, O26, and Cu^i^, O26^i^ atoms. The values of the two different lengths of the edges of the rectangular sides are 2.4280 (14) and 1.9684 (13) Å. The other faces of the two open-cubes are irregular with different distances *i.e.* Cu1—O26^i^ = 2.4280 (14) Å, Cu2—O26 = 1.9707 (14) Å, Cu1—Cl3 = 2.2181 (6) Å and Cu2—Cl3 = 2.8134 (6) Å. The Cu1 (Cu1^i^) atoms in each of the two CuO_3_NCl units are connected by one μ_2_-O and two μ_3_-O atoms from the deprotonated hydroxyl groups and one chloride ion to three other Cu^II^ cations. In the CuO_2_NCl_2_ units, the Cu2 (Cu2^i^) atoms are linked to one μ_2_-O and one μ_3_-O atoms from a deprotonated hydroxyl groups and one chloride ion to two other Cu^II^ cations with Cu1—Cu2 and Cu1—Cu2^i^ distances of approximately 3.012 and 3.408 Å, respectively. These are in good agreement with literature values (Qin *et al.*, 2014 ▸). The distances of the oxygen atoms in the μ_3_- and μ_2_-bridging positions to the copper atoms are assymmetrical with Cu1—O26^i^, Cu1—O26 and Cu2—O26 distances of 2.4280 (14), 1.9684 (13), 1.9707 (14) Å, respectively, while Cu1—O15 and Cu2—O15 are 1.9170 (13) and 1.9324 (13) Å, respectively (Table 4 ▸). These distances agree with those in related structures (Laza­rou *et al.*, 2018 ▸; Tabassum *et al.*, 2017 ▸). The environment of both Cu^II^ cations is again best described as distorted square pyramidal. The largest angles around Cu1 and Cu2 are O15—Cu1—Cl3 [176.95 (5)°], O26—Cu1—N10 [156.02 (7)°], O26—Cu2—Cl4 [170.05 (5)°] and O15—Cu2—N21 [157.61 (7)°] (Table 2 ▸). The Addison *τ* parameters are 0.348 for Cu1 and 0.207 for Cu2 (Addison *et al.*, 1984 ▸), indicating considerable distortion. The basal plane around each of the Cu1 and Cu2 atoms is formed by one chloride anion, one pyridine nitro­gen atom and two enolate oxygen atoms while the apical positions are occupied by an enolate oxygen atom for Cu1 and a chloride anion for Cu2. The copper–halogen distances Cu1—Cl3 and Cu2—Cl3 of 2.2181 (6) and 2.8134 (6) Å, respectively, agree with those for a chloride ion in bridging position (Choubey *et al.*, 2015 ▸). The Cu2—Cl4 distance of 2.1987 (7) Å is indicative of a unidentate terminal chloride ion (Kalinowska-Lis *et al.*, 2011 ▸). The four copper atoms occupy the vertices of a parallelogram with angles Cu1—Cu2—Cu1^i^ and Cu2—Cu1—Cu2^i^ of approximately 63.59° and 116.41°. The sum of the angle in the parallelogram is 360° and the lengths of the two diagonals, Cu1—Cu1^i^ and Cu2—Cu2^i^, are 3.399 and 5.461 Å respectively and are comparable to the values found in a similar complex reported in the literature (Monfared *et al.*, 2009 ▸). All the Cu—O—Cu angles in the open-cube are in the range 99.76 (6)—102.96 (6)° and the Cu1—Cl3—Cu2^i^ angles of 84.39 (2)° are different from those of ideal cube. This bridging angle is also smaller than those reported for similar complexes (Banerjee *et al.*, 2013 ▸; Swank *et al.*, 1979 ▸) but they are nearly equal to those in the complex [Cu_2_(qsalBr)_2_Cl_2_](DMF) where qsalBr = 8-amino­quinoline with 5-bromo-salicyl­aldehyde (Liu *et al.*, 2009 ▸). An immediate consequence is a small Cu1⋯Cu2 separation [3.4082 (4) Å] compared to those found in another di­chlorido-bridged copper (II) complex (Banerjee *et al.*, 2013 ▸).

## Supra­molecular features   

**Figure 6:**
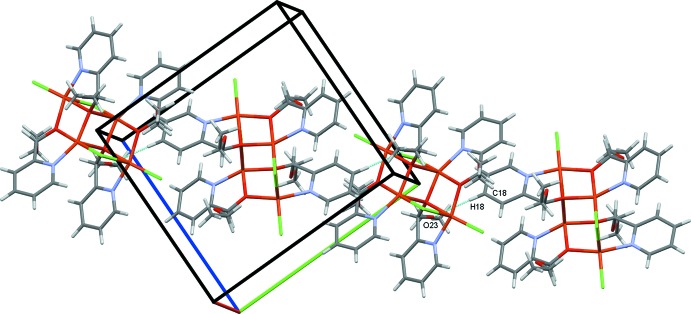
Chains of mol­ecules of **II** along the *bc* diagonal.

**Figure 7:**
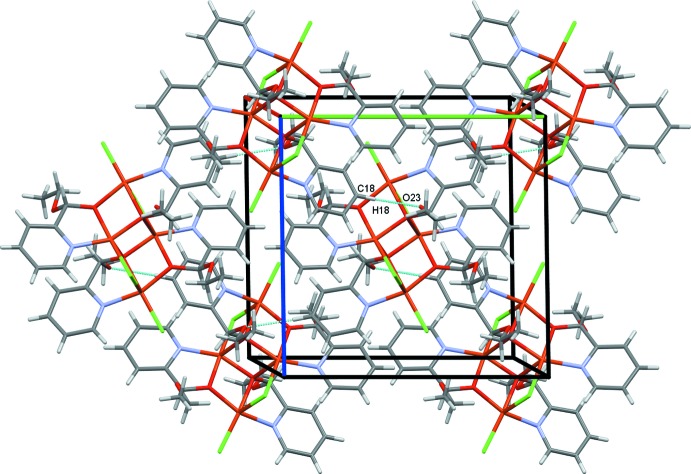
Two-dimensional sheet of mol­ecules of **II** along the *bc* diagonal.

**Figure 8:**
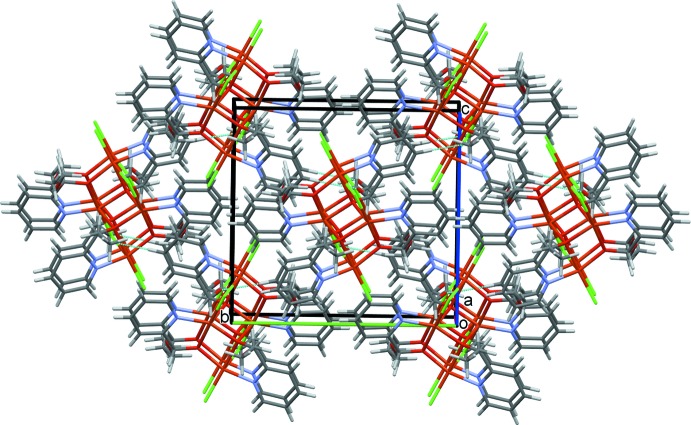
A view along the *a* axis of the crystal packing of **II**.

The crystal structure of **I** is determined by a coordination synthon in which each ligand is coordinated to two metal centers, giving rise to infinite one-dimensional polymeric chains along the *b-*axis direction (Fig. 2 ▸). Adjacent chains are linked to one another by inter­molecular C—H⋯O and C—H⋯Cl hydrogen bonds (Table 1 ▸), leading to a three-dimensional network structure (Fig. 3 ▸). In the crystal structure of **II**, C18—H18⋯O23 hydrogen bonds link the complex mol­ecules into chains along the *bc* diagonal (Fig. 6 ▸). Additional C18—H18⋯O23 contacts generate two-dimensional sheets of mol­ecules also along the *bc* diagonal (Fig. 7 ▸). π–π-stacking inter­actions occur between the two unique N10/C5–C9 and N21/C16–C20 pyridine rings with a centroid-to-centroid separation of 3.6800 (16) Å (symmetry operation 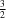 − *x*, −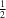 + *y*, 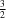 − *z*). These contacts combine with the C—H⋯O hydrogen bonds to stack the mol­ecules in a three-dimensional network along the *a*-axis direction (Fig. 8 ▸).

## Database survey   

A search of the CSD database (Version 5.38; Groom *et al.*, 2016 ▸) for the structures **I** and **II** using the fragment [1-eth­oxy-1-(pyridin-2­yl)]methyl­enehydrazine yielded no hits, indicating that compound **I** is reasonably unique. However, a search for eth­oxy(pyridin-2-yl)methano­late, the ligand found in **II** gave ten hits, although none of these was closely related to **II**. The matches included the Cu^II^ complexes HAXBEN (Baggio *et al.*, 1993 ▸), HUXDOU (Mautner *et al.*, 2010 ▸), TOGLAC (Deveson *et al.*, 1996 ▸) and VIMCAX (Efthymiou *et al.*, 2013 ▸) that involve the eth­oxy­dipyridin-2-yl­methanol ligand, which differs from the ligand reported here by substitution of the hydrogen atom on the carbon of the alcohol unit by a pyridine ring. A similar substitution with phenyl or by a 2-hy­droxy­pyridine ring leads to the Cu^II^ complexes JUYYEJ (Kitos *et al.*, 2016 ▸) and COHQIA (Boudalis *et al.*, 2008 ▸), respectively. The three related hits QANPUQ, QANQAX and QANQEB (Papaefsta­thiou *et al.*, 2000 ▸) are complexes of the symmetrical ligand 1,2-dieth­oxy-1,2-di(pyridin-2-yl)ethane-1,2-diol, which is a dimer of the ligand found in **II**. KAJKAJ (Georgopoulou *et al.*, 2010 ▸) involves a Cu complex of a ligand that is the least similar to that found in **II**. The ligand used, 2,6-bis­[1-eth­oxy-1-hy­droxy-1-(pyridin-2-yl)meth­yl]pyridin, has a central pyridine ring that is substituted by 1-eth­oxy-1-hy­droxy-1-(pyridin-2-yl)methyl fragments in the 2- and 6-positions.

## Synthesis and crystallization   

To a solution of 2-pyridine carbaldehyde (0.1070 g, 1 mmol) in 30 ml of ethanol was added a solution of nicotinic hydrazide (0.1371 g, 1 mmol) in 10 ml of ethanol. The mixture was stirred for 5 min. A solution of Cu(OOCH_3_)_2_·H_2_O (0.1996 g, 1 mmol) in 5 ml of ethanol was added at room temperature. The initial yellow solution immediately turned deep blue and was stirred under reflux for 2 h. The mixture was filtered and the solution evaporated to near dryness. The solid was isolated by filtration and recrystallized from a minimum of ethanol. On standing for five days, two types of crystals suitable for X-ray analysis were formed, light-yellow blocks of **I** and light-green plates of **II**.

For **I**: analysis calculated: C_14_H_13_N_4_ClO_2_Cu: C, 45.46; H, 3.56; N, 15.21; Cl; 9.63. Found: C, 45.44; H, 3.53; N, 15.16; Cl; 9.60. IR (ν, cm^−1^): 2982, 1628, 1583, 1423, 1343, 1245, 941, 816, 630. For **II:** analysis calculated: C_16_H_20_N_2_Cl_2_O_4_Cu_2_: C, 38.26; H, 4.01; N, 5.58; Cl; 14.12. Found: C, 38.23; H, 3.98; N, 5.55; Cl; 14.08. IR (ν, cm^−1^): 2982, 1585, 1423, 1243, 1145, 940, 812.

## Refinement   

Crystal data, data collection and structure refinement details are summarized in Table 5 ▸. All H atoms were refined using a riding model with *d*(C—H) = 0.93 Å for aromatic, *d*(C—H) = 0.97 Å for methyl­ene and *d*(C—H) = 0.98 Å for methine H atoms with *U*_iso_(H) = 1.2*U*_eq_(C) and *d*(C—H) = 0.96 Å and *U*_iso_(H) = 1.5*U*_eq_(C) for methyl H atoms. One reflection with *F*_o_ <<< *F*_c_ that was likely to have been affected by the beamstop was omitted from the final refinement cycles.

## Supplementary Material

Crystal structure: contains datablock(s) I, II. DOI: 10.1107/S2056989019008922/sj5575sup1.cif

Structure factors: contains datablock(s) I. DOI: 10.1107/S2056989019008922/sj5575Isup4.hkl

Structure factors: contains datablock(s) II. DOI: 10.1107/S2056989019008922/sj5575IIsup5.hkl

CCDC references: 1935842, 1935841

Additional supporting information:  crystallographic information; 3D view; checkCIF report

## Figures and Tables

**Table 1:** Hydrogen-bond geometry (Å, °) for **I** [Chem scheme1]

*D*—H⋯*A*	*D*—H	H⋯*A*	*D*⋯*A*	*D*—H⋯*A*
C8—H8⋯Cl2^ii^	0.93	2.71	3.354 (2)	128
C20—H20⋯Cl2^iii^	0.93	2.93	3.527 (3)	124
C14—H14*B*⋯Cl2^iv^	0.97	2.83	3.534 (3)	130
C14—H14*B*⋯O16^iv^	0.97	2.56	3.441 (3)	151

Symmetry codes: (ii) 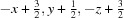; (iii) 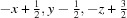; (iv) 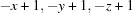.

**Table 2:** Selected geometric parameters (Å, °) for **I** [Chem scheme1]

Cu1—N11	1.9437 (17)	Cu1—N3^i^	2.2009 (17)
Cu1—O16	1.9808 (15)	Cu1—Cl2	2.2707 (6)
Cu1—N22	2.0444 (17)		
			
N11—Cu1—O16	79.11 (7)	O16—Cu1—N3^i^	96.39 (7)
N11—Cu1—N22	79.40 (7)	N22—Cu1—N3^i^	92.70 (7)
O16—Cu1—N22	158.51 (7)	N11—Cu1—Cl2	146.17 (6)
N11—Cu1—N3^i^	116.09 (7)	O16—Cu1—Cl2	100.05 (5)

Symmetry code: (i) 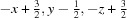.

**Table 3:** Hydrogen-bond geometry (Å, °) for **II** [Chem scheme1]

*D*—H⋯*A*	*D*—H	H⋯*A*	*D*⋯*A*	*D*—H⋯*A*
C9—H9⋯Cl3	0.93	2.78	3.333 (3)	119
C20—H20⋯Cl4	0.93	2.90	3.393 (3)	115
C13—H13*B*⋯Cl3^i^	0.97	2.82	3.787 (3)	173
C22—H22⋯O12^i^	0.98	2.66	3.578 (3)	156
C18—H18⋯O23^ii^	0.93	2.44	3.367 (3)	177

Symmetry codes: (i) 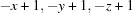; (ii) 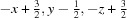.

**Table 4:** Selected geometric parameters (Å, °) for **II**)

Cu1—O15	1.9170 (13)	Cu2—O15	1.9324 (13)
Cu1—O26	1.9684 (13)	Cu2—O26	1.9707 (14)
Cu1—N10	1.9886 (17)	Cu2—N21	1.9827 (17)
Cu1—Cl3	2.2181 (6)	Cu2—Cl4	2.1987 (7)
Cu1—O26^i^	2.4280 (14)		
			
O26—Cu1—N10	156.02 (7)	O15—Cu2—N21	157.61 (7)
O15—Cu1—Cl3	176.95 (5)	O26—Cu2—Cl4	170.05 (5)

Symmetry code: (i) 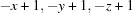.

**Table 5:** Experimental details

	**I**	**II**
Crystal data		
Chemical formula	[Cu(C_14_H_13_N_4_O_2_)Cl]	[Cu_4_(C_8_H_10_NO_2_)_4_Cl_4_]
*M*_r_	368.27	1004.68
Crystal system, space group	Monoclinic, *P*2_1_/*n*	Monoclinic, *P*2_1_/*n*
Temperature (K)	293	293
*a*, *b*, *c* (Å)	11.1472 (9), 9.9573 (6), 14.4904 (11)	11.5150 (4), 13.1051 (5), 12.8066 (6)
β (°)	111.595 (9)	100.066 (4)
*V* (Å^3^)	1495.5 (2)	1902.83 (13)
*Z*	4	2
Radiation type	Mo *K*α	Mo *K*α
μ (mm^−1^)	1.65	2.54
Crystal size (mm)	0.3 × 0.2 × 0.1	0.22 × 0.2 × 0.05

Data collection		
Diffractometer	Rigaku Oxford Diffraction XtaLAB Mini (ROW)	Rigaku Oxford Diffraction XtaLAB Mini (ROW)
Absorption correction	Multi-scan (*CrysAlis PRO*; Rigaku OD, 2017 ▸)	Multi-scan (*CrysAlis PRO*; Rigaku OD, 2017 ▸)
*T*_min_, *T*_max_	0.967, 1.000	0.727, 1.000
No. of measured, independent and observed [*I* > 2σ(*I*)] reflections	8476, 5569, 3671	31609, 7541, 4946
*R*_int_	0.021	0.035
(sin θ/λ)_max_ (Å^−1^)	0.797	0.797

Refinement		
*R*[*F* ^2^ > 2σ(*F* ^2^)], *wR*(*F* ^2^), *S*	0.041, 0.109, 1.02	0.035, 0.093, 1.02
No. of reflections	5569	7540
No. of parameters	200	237
H-atom treatment	H-atom parameters constrained	H-atom parameters constrained
Δρ_max_, Δρ_min_ (e Å^−3^)	0.40, −0.42	0.46, −0.43

Computer programs: *CrysAlis PRO* (Rigaku OD, 2017 ▸), *SHELXT2018/3* (Sheldrick, 2015*a* ▸), *SHELXL2018/3* (Sheldrick, 2015*b* ▸), *OLEX2* (Dolomanov *et al.*, 2009 ▸) and *Mercury* (Macrae *et al.*, 2008 ▸).

## supplementary crystallographic information

### catena-Poly[[chloridocopper(II)]-µ-N-[ethoxy(pyridin-2-yl)methylidene]-N'-[oxido(pyridin-3-yl)methylidene]hydrazine-κ4N,N',O:N''] (I) Crystal data

**Table:**

[Cu(C_14_H_13_N_4_O_2_)Cl]	*F*(000) = 748
*M**_r_* = 368.27	*D*_x_ = 1.636 Mg m^−^^3^
Monoclinic, *P*2_1_/*n*	Mo *K*α radiation, λ = 0.71073 Å
*a* = 11.1472 (9) Å	Cell parameters from 2449 reflections
*b* = 9.9573 (6) Å	θ = 3.5–28.8°
*c* = 14.4904 (11) Å	µ = 1.65 mm^−^^1^
β = 111.595 (9)°	*T* = 293 K
*V* = 1495.5 (2) Å^3^	Block, clear light yellow
*Z* = 4	0.3 × 0.2 × 0.1 mm

### catena-Poly[[chloridocopper(II)]-µ-N-[ethoxy(pyridin-2-yl)methylidene]-N'-[oxido(pyridin-3-yl)methylidene]hydrazine-κ4N,N',O:N''] (I) Data collection

**Table:**

Rigaku Oxford Diffraction XtaLAB Mini (ROW) diffractometer	5569 independent reflections
Radiation source: fine-focus sealed X-ray tube, Rigaku (Mo) X-ray Source	3671 reflections with *I* > 2σ(*I*)
Graphite monochromator	*R*_int_ = 0.021
ω scans	θ_max_ = 34.5°, θ_min_ = 2.8°
Absorption correction: multi-scan (CrysAlisPro; Rigaku OD, 2017)	*h* = −5→17
*T*_min_ = 0.967, *T*_max_ = 1.000	*k* = −14→12
8476 measured reflections	*l* = −21→20

### catena-Poly[[chloridocopper(II)]-µ-N-[ethoxy(pyridin-2-yl)methylidene]-N'-[oxido(pyridin-3-yl)methylidene]hydrazine-κ4N,N',O:N''] (I) Refinement

**Table:**

Refinement on *F*^2^	0 restraints
Least-squares matrix: full	Hydrogen site location: inferred from neighbouring sites
*R*[*F*^2^ > 2σ(*F*^2^)] = 0.041	H-atom parameters constrained
*wR*(*F*^2^) = 0.109	*w* = 1/[σ^2^(*F*_o_^2^) + (0.0492*P*)^2^ + 0.1803*P*] where *P* = (*F*_o_^2^ + 2*F*_c_^2^)/3
*S* = 1.02	(Δ/σ)_max_ = 0.001
5569 reflections	Δρ_max_ = 0.40 e Å^−^^3^
200 parameters	Δρ_min_ = −0.41 e Å^−^^3^

### catena-Poly[[chloridocopper(II)]-µ-N-[ethoxy(pyridin-2-yl)methylidene]-N'-[oxido(pyridin-3-yl)methylidene]hydrazine-κ4N,N',O:N''] (I) Special details

**Table:**

Geometry. All esds (except the esd in the dihedral angle between two l.s. planes) are estimated using the full covariance matrix. The cell esds are taken into account individually in the estimation of esds in distances, angles and torsion angles; correlations between esds in cell parameters are only used when they are defined by crystal symmetry. An approximate (isotropic) treatment of cell esds is used for estimating esds involving l.s. planes.

### catena-Poly[[chloridocopper(II)]-µ-N-[ethoxy(pyridin-2-yl)methylidene]-N'-[oxido(pyridin-3-yl)methylidene]hydrazine-κ4N,N',O:N''] (I) Fractional atomic coordinates and isotropic or equivalent isotropic displacement parameters (Å^2^)

**Table:**

	*x*	*y*	*z*	*U*_iso_*/*U*_eq_	
Cu1	0.47474 (2)	0.32922 (3)	0.65558 (2)	0.03498 (9)	
Cl2	0.34273 (6)	0.44640 (6)	0.71389 (4)	0.04555 (14)	
O16	0.61729 (15)	0.46011 (15)	0.68180 (11)	0.0409 (3)	
N11	0.52597 (16)	0.29579 (17)	0.54312 (13)	0.0351 (4)	
O13	0.47178 (19)	0.17375 (17)	0.39271 (13)	0.0548 (5)	
N3	0.93076 (16)	0.70447 (17)	0.71207 (13)	0.0347 (4)	
N22	0.34839 (17)	0.17999 (17)	0.58487 (13)	0.0360 (4)	
N10	0.62800 (18)	0.3710 (2)	0.53850 (14)	0.0408 (4)	
C5	0.77400 (18)	0.5424 (2)	0.62038 (14)	0.0316 (4)	
C9	0.66495 (18)	0.4523 (2)	0.61446 (14)	0.0325 (4)	
C17	0.3576 (2)	0.1438 (2)	0.49841 (15)	0.0350 (4)	
C4	0.82919 (19)	0.6265 (2)	0.70085 (15)	0.0337 (4)	
H4	0.793616	0.628682	0.749681	0.040*	
C12	0.4615 (2)	0.2121 (2)	0.47694 (15)	0.0356 (4)	
C6	0.8249 (2)	0.5432 (2)	0.54629 (16)	0.0392 (4)	
H6	0.789474	0.489398	0.490347	0.047*	
C8	0.9789 (2)	0.7018 (2)	0.64056 (16)	0.0414 (5)	
H8	1.050395	0.754901	0.647646	0.050*	
C7	0.9278 (2)	0.6241 (3)	0.55673 (17)	0.0450 (5)	
H7	0.962947	0.626743	0.507670	0.054*	
C18	0.2755 (2)	0.0507 (2)	0.43614 (18)	0.0479 (6)	
H18	0.281599	0.028794	0.375622	0.057*	
C21	0.2599 (2)	0.1209 (3)	0.61202 (17)	0.0455 (5)	
H21	0.253554	0.145443	0.671970	0.055*	
C20	0.1774 (3)	0.0248 (3)	0.5549 (2)	0.0551 (6)	
H20	0.117711	−0.016619	0.576519	0.066*	
C14	0.5530 (3)	0.2442 (3)	0.34999 (18)	0.0490 (6)	
H14A	0.643386	0.228648	0.389388	0.059*	
H14B	0.536575	0.339998	0.347922	0.059*	
C19	0.1847 (3)	−0.0087 (3)	0.4658 (2)	0.0573 (7)	
H19	0.128097	−0.071808	0.425264	0.069*	
C15	0.5207 (3)	0.1908 (3)	0.2480 (2)	0.0725 (9)	
H15A	0.429643	0.199448	0.211706	0.109*	
H15B	0.544680	0.097758	0.251411	0.109*	
H15C	0.566903	0.240523	0.214954	0.109*	

### catena-Poly[[chloridocopper(II)]-µ-N-[ethoxy(pyridin-2-yl)methylidene]-N'-[oxido(pyridin-3-yl)methylidene]hydrazine-κ4N,N',O:N''] (I) Atomic displacement parameters (Å^2^)

**Table:**

	*U*^11^	*U*^22^	*U*^33^	*U*^12^	*U*^13^	*U*^23^
Cu1	0.03827 (14)	0.04034 (15)	0.02896 (13)	−0.00397 (10)	0.01546 (10)	−0.00355 (11)
Cl2	0.0534 (3)	0.0477 (3)	0.0432 (3)	0.0115 (2)	0.0267 (3)	0.0022 (2)
O16	0.0442 (8)	0.0487 (8)	0.0340 (7)	−0.0114 (7)	0.0194 (6)	−0.0095 (7)
N11	0.0368 (8)	0.0381 (9)	0.0340 (8)	−0.0065 (7)	0.0173 (7)	−0.0053 (7)
O13	0.0726 (12)	0.0579 (11)	0.0474 (10)	−0.0254 (9)	0.0381 (9)	−0.0184 (8)
N3	0.0373 (8)	0.0373 (9)	0.0298 (8)	−0.0047 (7)	0.0126 (7)	−0.0014 (7)
N22	0.0375 (8)	0.0412 (9)	0.0303 (8)	−0.0038 (7)	0.0138 (7)	0.0020 (7)
N10	0.0422 (9)	0.0468 (10)	0.0380 (10)	−0.0118 (8)	0.0201 (8)	−0.0099 (8)
C5	0.0321 (9)	0.0334 (9)	0.0275 (9)	0.0014 (7)	0.0089 (7)	0.0013 (8)
C9	0.0333 (9)	0.0349 (9)	0.0283 (9)	0.0006 (8)	0.0103 (8)	0.0011 (8)
C17	0.0403 (10)	0.0331 (10)	0.0324 (10)	−0.0021 (8)	0.0142 (8)	0.0012 (8)
C4	0.0369 (10)	0.0363 (10)	0.0294 (9)	−0.0006 (8)	0.0139 (8)	0.0004 (8)
C12	0.0410 (10)	0.0380 (10)	0.0319 (10)	−0.0045 (8)	0.0182 (8)	−0.0034 (8)
C6	0.0435 (11)	0.0439 (11)	0.0297 (10)	−0.0041 (9)	0.0129 (9)	−0.0045 (9)
C8	0.0451 (12)	0.0464 (12)	0.0367 (11)	−0.0099 (9)	0.0197 (9)	−0.0042 (9)
C7	0.0526 (13)	0.0542 (13)	0.0357 (11)	−0.0082 (11)	0.0250 (10)	−0.0049 (10)
C18	0.0556 (14)	0.0505 (13)	0.0389 (12)	−0.0151 (11)	0.0189 (11)	−0.0105 (11)
C21	0.0471 (12)	0.0583 (14)	0.0352 (11)	−0.0097 (11)	0.0198 (10)	0.0025 (11)
C20	0.0527 (14)	0.0652 (16)	0.0499 (14)	−0.0203 (12)	0.0219 (12)	0.0025 (13)
C14	0.0583 (14)	0.0520 (14)	0.0460 (13)	−0.0135 (11)	0.0299 (11)	−0.0080 (11)
C19	0.0589 (15)	0.0622 (16)	0.0515 (15)	−0.0280 (13)	0.0212 (13)	−0.0107 (13)
C15	0.091 (2)	0.087 (2)	0.0555 (16)	−0.0389 (18)	0.0467 (16)	−0.0246 (16)

### catena-Poly[[chloridocopper(II)]-µ-N-[ethoxy(pyridin-2-yl)methylidene]-N'-[oxido(pyridin-3-yl)methylidene]hydrazine-κ4N,N',O:N''] (I) Geometric parameters (Å, º)

**Table:**

Cu1—N11	1.9437 (17)	C4—H4	0.9300
Cu1—O16	1.9808 (15)	C6—C7	1.363 (3)
Cu1—N22	2.0444 (17)	C6—H6	0.9300
Cu1—N3^i^	2.2009 (17)	C8—C7	1.374 (3)
Cu1—Cl2	2.2707 (6)	C8—H8	0.9300
O16—C9	1.274 (2)	C7—H7	0.9300
N11—C12	1.274 (3)	C18—C19	1.371 (3)
N11—N10	1.384 (2)	C18—H18	0.9300
O13—C12	1.323 (3)	C21—C20	1.374 (3)
O13—C14	1.452 (3)	C21—H21	0.9300
N3—C8	1.331 (3)	C20—C19	1.364 (4)
N3—C4	1.333 (3)	C20—H20	0.9300
N22—C21	1.326 (3)	C14—C15	1.485 (3)
N22—C17	1.344 (3)	C14—H14A	0.9700
N10—C9	1.305 (3)	C14—H14B	0.9700
C5—C4	1.382 (3)	C19—H19	0.9300
C5—C6	1.387 (3)	C15—H15A	0.9600
C5—C9	1.488 (3)	C15—H15B	0.9600
C17—C18	1.380 (3)	C15—H15C	0.9600
C17—C12	1.472 (3)		
			
N11—Cu1—O16	79.11 (7)	N11—C12—C17	114.38 (18)
N11—Cu1—N22	79.40 (7)	O13—C12—C17	113.79 (18)
O16—Cu1—N22	158.51 (7)	C7—C6—C5	119.0 (2)
N11—Cu1—N3^i^	116.09 (7)	C7—C6—H6	120.5
O16—Cu1—N3^i^	96.39 (7)	C5—C6—H6	120.5
N22—Cu1—N3^i^	92.70 (7)	N3—C8—C7	123.0 (2)
N11—Cu1—Cl2	146.17 (6)	N3—C8—H8	118.5
O16—Cu1—Cl2	100.05 (5)	C7—C8—H8	118.5
N22—Cu1—Cl2	97.97 (5)	C6—C7—C8	119.3 (2)
N3^i^—Cu1—Cl2	97.68 (5)	C6—C7—H7	120.4
C9—O16—Cu1	110.13 (13)	C8—C7—H7	120.4
C12—N11—N10	124.29 (17)	C19—C18—C17	118.3 (2)
C12—N11—Cu1	119.01 (14)	C19—C18—H18	120.9
N10—N11—Cu1	116.63 (13)	C17—C18—H18	120.9
C12—O13—C14	122.21 (18)	N22—C21—C20	122.4 (2)
C8—N3—C4	117.47 (18)	N22—C21—H21	118.8
C8—N3—Cu1^ii^	119.26 (14)	C20—C21—H21	118.8
C4—N3—Cu1^ii^	123.23 (14)	C19—C20—C21	118.7 (2)
C21—N22—C17	118.57 (19)	C19—C20—H20	120.6
C21—N22—Cu1	128.61 (16)	C21—C20—H20	120.6
C17—N22—Cu1	112.78 (13)	O13—C14—C15	106.9 (2)
C9—N10—N11	107.74 (17)	O13—C14—H14A	110.3
C4—C5—C6	117.88 (19)	C15—C14—H14A	110.3
C4—C5—C9	120.96 (18)	O13—C14—H14B	110.3
C6—C5—C9	121.15 (18)	C15—C14—H14B	110.3
O16—C9—N10	126.39 (19)	H14A—C14—H14B	108.6
O16—C9—C5	118.76 (17)	C20—C19—C18	119.9 (2)
N10—C9—C5	114.86 (18)	C20—C19—H19	120.0
N22—C17—C18	122.0 (2)	C18—C19—H19	120.0
N22—C17—C12	114.18 (18)	C14—C15—H15A	109.5
C18—C17—C12	123.8 (2)	C14—C15—H15B	109.5
N3—C4—C5	123.38 (19)	H15A—C15—H15B	109.5
N3—C4—H4	118.3	C14—C15—H15C	109.5
C5—C4—H4	118.3	H15A—C15—H15C	109.5
N11—C12—O13	131.82 (19)	H15B—C15—H15C	109.5

Symmetry codes: (i) −*x*+3/2, *y*−1/2, −*z*+3/2; (ii) −*x*+3/2, *y*+1/2, −*z*+3/2.

### catena-Poly[[chloridocopper(II)]-µ-N-[ethoxy(pyridin-2-yl)methylidene]-N'-[oxido(pyridin-3-yl)methylidene]hydrazine-κ4N,N',O:N''] (I) Hydrogen-bond geometry (Å, º)

**Table:**

*D*—H···*A*	*D*—H	H···*A*	*D*···*A*	*D*—H···*A*
C8—H8···Cl2^ii^	0.93	2.71	3.354 (2)	128
C20—H20···Cl2^iii^	0.93	2.93	3.527 (3)	124
C14—H14*B*···Cl2^iv^	0.97	2.83	3.534 (3)	130
C14—H14*B*···O16^iv^	0.97	2.56	3.441 (3)	151

Symmetry codes: (ii) −*x*+3/2, *y*+1/2, −*z*+3/2; (iii) −*x*+1/2, *y*−1/2, −*z*+3/2; (iv) −*x*+1, −*y*+1, −*z*+1.

### Di-µ-chlorido-1:4κ2Cl:Cl-2:3κ2Cl:Cl-dichlorido-2κCl,4κCl-bis[µ3-ethoxy(pyridin-2-yl)methanolato-1:2:3κ3O:N,O:O;1:3:4κ3O:O:N,O]bis[µ2-ethoxy(pyridin-2-yl)methanolato-1:2κ3N,O:O;3:4κ3N,O:O]tetracopper(II) (II) Crystal data

**Table:**

[Cu_4_(C_8_H_10_NO_2_)_4_Cl_4_]	*F*(000) = 1016
*M**_r_* = 1004.68	*D*_x_ = 1.753 Mg m^−^^3^
Monoclinic, *P*2_1_/*n*	Mo *K*α radiation, λ = 0.71073 Å
*a* = 11.5150 (4) Å	Cell parameters from 9290 reflections
*b* = 13.1051 (5) Å	θ = 3.0–32.4°
*c* = 12.8066 (6) Å	µ = 2.54 mm^−^^1^
β = 100.066 (4)°	*T* = 293 K
*V* = 1902.83 (13) Å^3^	Plate, clear light green
*Z* = 2	0.22 × 0.2 × 0.05 mm

### Di-µ-chlorido-1:4κ2Cl:Cl-2:3κ2Cl:Cl-dichlorido-2κCl,4κCl-bis[µ3-ethoxy(pyridin-2-yl)methanolato-1:2:3κ3O:N,O:O;1:3:4κ3O:O:N,O]bis[µ2-ethoxy(pyridin-2-yl)methanolato-1:2κ3N,O:O;3:4κ3N,O:O]tetracopper(II) (II) Data collection

**Table:**

Rigaku Oxford Diffraction XtaLAB Mini (ROW) diffractometer	7541 independent reflections
Radiation source: fine-focus sealed X-ray tube, Rigaku (Mo) X-ray Source	4946 reflections with *I* > 2σ(*I*)
Graphite monochromator	*R*_int_ = 0.035
ω scans	θ_max_ = 34.5°, θ_min_ = 2.6°
Absorption correction: multi-scan (CrysAlisPro; Rigaku OD, 2017)	*h* = −17→18
*T*_min_ = 0.727, *T*_max_ = 1.000	*k* = −20→14
31609 measured reflections	*l* = −18→18

### Di-µ-chlorido-1:4κ2Cl:Cl-2:3κ2Cl:Cl-dichlorido-2κCl,4κCl-bis[µ3-ethoxy(pyridin-2-yl)methanolato-1:2:3κ3O:N,O:O;1:3:4κ3O:O:N,O]bis[µ2-ethoxy(pyridin-2-yl)methanolato-1:2κ3N,O:O;3:4κ3N,O:O]tetracopper(II) (II) Refinement

**Table:**

Refinement on *F*^2^	0 restraints
Least-squares matrix: full	Hydrogen site location: inferred from neighbouring sites
*R*[*F*^2^ > 2σ(*F*^2^)] = 0.035	H-atom parameters constrained
*wR*(*F*^2^) = 0.093	*w* = 1/[σ^2^(*F*_o_^2^) + (0.0422*P*)^2^ + 0.4202*P*] where *P* = (*F*_o_^2^ + 2*F*_c_^2^)/3
*S* = 1.02	(Δ/σ)_max_ = 0.001
7540 reflections	Δρ_max_ = 0.46 e Å^−^^3^
237 parameters	Δρ_min_ = −0.43 e Å^−^^3^

### Di-µ-chlorido-1:4κ2Cl:Cl-2:3κ2Cl:Cl-dichlorido-2κCl,4κCl-bis[µ3-ethoxy(pyridin-2-yl)methanolato-1:2:3κ3O:N,O:O;1:3:4κ3O:O:N,O]bis[µ2-ethoxy(pyridin-2-yl)methanolato-1:2κ3N,O:O;3:4κ3N,O:O]tetracopper(II) (II) Special details

**Table:**

Geometry. All esds (except the esd in the dihedral angle between two l.s. planes) are estimated using the full covariance matrix. The cell esds are taken into account individually in the estimation of esds in distances, angles and torsion angles; correlations between esds in cell parameters are only used when they are defined by crystal symmetry. An approximate (isotropic) treatment of cell esds is used for estimating esds involving l.s. planes.

### Di-µ-chlorido-1:4κ2Cl:Cl-2:3κ2Cl:Cl-dichlorido-2κCl,4κCl-bis[µ3-ethoxy(pyridin-2-yl)methanolato-1:2:3κ3O:N,O:O;1:3:4κ3O:O:N,O]bis[µ2-ethoxy(pyridin-2-yl)methanolato-1:2κ3N,O:O;3:4κ3N,O:O]tetracopper(II) (II) Fractional atomic coordinates and isotropic or equivalent isotropic displacement parameters (Å^2^)

**Table:**

	*x*	*y*	*z*	*U*_iso_*/*U*_eq_	
Cu1	0.59802 (2)	0.59673 (2)	0.49846 (2)	0.03411 (7)	
Cu2	0.55029 (2)	0.53449 (2)	0.71340 (2)	0.03564 (7)	
Cl3	0.67941 (4)	0.53396 (4)	0.36744 (4)	0.04527 (12)	
Cl4	0.53662 (7)	0.61216 (5)	0.86295 (5)	0.06187 (17)	
O15	0.53512 (12)	0.64894 (10)	0.61683 (11)	0.0386 (3)	
O26	0.59121 (12)	0.47291 (10)	0.58419 (11)	0.0370 (3)	
O12	0.40663 (13)	0.78727 (11)	0.59492 (12)	0.0454 (3)	
O23	0.78892 (13)	0.44737 (12)	0.63858 (14)	0.0503 (4)	
N21	0.60042 (14)	0.39764 (13)	0.77014 (15)	0.0418 (4)	
N10	0.61957 (15)	0.74464 (13)	0.47288 (14)	0.0410 (4)	
C5	0.58862 (18)	0.80472 (15)	0.54740 (18)	0.0409 (4)	
C11	0.52350 (17)	0.75255 (15)	0.62500 (17)	0.0381 (4)	
H11	0.555202	0.774842	0.697424	0.046*	
C16	0.65162 (16)	0.34167 (15)	0.70334 (18)	0.0423 (5)	
C22	0.67794 (16)	0.39909 (14)	0.60830 (17)	0.0385 (4)	
H22	0.678628	0.353075	0.548094	0.046*	
C17	0.6800 (2)	0.24007 (17)	0.7229 (2)	0.0581 (6)	
H17	0.714861	0.202010	0.675423	0.070*	
C20	0.5775 (2)	0.3558 (2)	0.8600 (2)	0.0551 (6)	
H20	0.543030	0.395046	0.906778	0.066*	
C13	0.3299 (2)	0.7534 (2)	0.6640 (2)	0.0521 (5)	
H13A	0.360939	0.773547	0.736401	0.062*	
H13B	0.323321	0.679615	0.661564	0.062*	
C18	0.6556 (2)	0.1970 (2)	0.8141 (3)	0.0747 (9)	
H18	0.673261	0.128697	0.828962	0.090*	
C9	0.6739 (2)	0.78622 (19)	0.3992 (2)	0.0555 (6)	
H9	0.695863	0.744342	0.347368	0.067*	
C6	0.6124 (2)	0.90782 (17)	0.5516 (2)	0.0579 (6)	
H6	0.591579	0.948143	0.605219	0.069*	
C19	0.6050 (2)	0.2547 (2)	0.8833 (3)	0.0725 (9)	
H19	0.589245	0.226032	0.945725	0.087*	
C24	0.8820 (2)	0.4028 (2)	0.5958 (2)	0.0584 (6)	
H24A	0.866484	0.409094	0.519149	0.070*	
H24B	0.888107	0.330848	0.613617	0.070*	
C7	0.6680 (3)	0.95004 (19)	0.4743 (3)	0.0725 (8)	
H7	0.684357	1.019542	0.474791	0.087*	
C8	0.6984 (3)	0.8886 (2)	0.3974 (3)	0.0692 (8)	
H8	0.735208	0.915673	0.344567	0.083*	
C14	0.2125 (2)	0.8005 (2)	0.6285 (2)	0.0666 (7)	
H14A	0.186088	0.785882	0.554729	0.100*	
H14B	0.218259	0.873080	0.638596	0.100*	
H14C	0.157202	0.773196	0.669194	0.100*	
C25	0.9948 (2)	0.4557 (3)	0.6401 (3)	0.0798 (9)	
H25A	1.059000	0.422938	0.614746	0.120*	
H25B	1.007511	0.452442	0.716177	0.120*	
H25C	0.990264	0.525820	0.618003	0.120*	

### Di-µ-chlorido-1:4κ2Cl:Cl-2:3κ2Cl:Cl-dichlorido-2κCl,4κCl-bis[µ3-ethoxy(pyridin-2-yl)methanolato-1:2:3κ3O:N,O:O;1:3:4κ3O:O:N,O]bis[µ2-ethoxy(pyridin-2-yl)methanolato-1:2κ3N,O:O;3:4κ3N,O:O]tetracopper(II) (II) Atomic displacement parameters (Å^2^)

**Table:**

	*U*^11^	*U*^22^	*U*^33^	*U*^12^	*U*^13^	*U*^23^
Cu1	0.03381 (11)	0.03383 (12)	0.03553 (13)	−0.00077 (8)	0.00838 (9)	0.00260 (9)
Cu2	0.03732 (12)	0.03519 (12)	0.03414 (13)	−0.00004 (9)	0.00550 (9)	0.00475 (9)
Cl3	0.0420 (2)	0.0496 (3)	0.0477 (3)	−0.0006 (2)	0.0175 (2)	−0.0010 (2)
Cl4	0.0881 (5)	0.0601 (4)	0.0372 (3)	−0.0049 (3)	0.0104 (3)	−0.0043 (3)
O15	0.0476 (7)	0.0318 (6)	0.0389 (7)	0.0029 (5)	0.0149 (6)	0.0051 (5)
O26	0.0372 (6)	0.0343 (6)	0.0399 (7)	0.0060 (5)	0.0081 (6)	0.0062 (5)
O12	0.0410 (7)	0.0460 (8)	0.0517 (9)	0.0077 (6)	0.0150 (7)	0.0080 (7)
O23	0.0342 (7)	0.0515 (9)	0.0674 (11)	−0.0063 (6)	0.0150 (7)	−0.0100 (8)
N21	0.0325 (8)	0.0443 (9)	0.0460 (10)	−0.0026 (7)	−0.0003 (7)	0.0135 (8)
N10	0.0453 (9)	0.0367 (8)	0.0426 (10)	−0.0035 (7)	0.0123 (7)	0.0045 (7)
C5	0.0393 (9)	0.0357 (9)	0.0488 (12)	−0.0004 (7)	0.0101 (8)	0.0052 (8)
C11	0.0383 (9)	0.0359 (9)	0.0411 (11)	0.0021 (7)	0.0098 (8)	0.0013 (8)
C16	0.0269 (8)	0.0386 (10)	0.0575 (13)	−0.0015 (7)	−0.0040 (8)	0.0096 (9)
C22	0.0311 (8)	0.0343 (9)	0.0489 (12)	0.0014 (7)	0.0032 (8)	−0.0001 (8)
C17	0.0393 (11)	0.0399 (11)	0.093 (2)	0.0034 (9)	0.0047 (12)	0.0143 (12)
C20	0.0395 (11)	0.0660 (15)	0.0574 (14)	−0.0040 (10)	0.0022 (10)	0.0271 (12)
C13	0.0453 (11)	0.0615 (14)	0.0524 (14)	0.0019 (10)	0.0167 (10)	0.0075 (11)
C18	0.0442 (13)	0.0533 (14)	0.122 (3)	0.0035 (11)	0.0014 (15)	0.0442 (17)
C9	0.0680 (15)	0.0502 (13)	0.0547 (14)	−0.0039 (11)	0.0282 (12)	0.0074 (11)
C6	0.0669 (15)	0.0369 (11)	0.0760 (18)	−0.0008 (10)	0.0297 (14)	0.0012 (11)
C19	0.0481 (13)	0.0775 (19)	0.089 (2)	−0.0041 (13)	0.0039 (13)	0.0503 (17)
C24	0.0408 (11)	0.0664 (16)	0.0699 (17)	0.0078 (10)	0.0151 (11)	0.0060 (13)
C7	0.090 (2)	0.0383 (12)	0.099 (2)	−0.0064 (12)	0.0429 (18)	0.0112 (13)
C8	0.084 (2)	0.0541 (15)	0.078 (2)	−0.0072 (13)	0.0398 (16)	0.0180 (14)
C14	0.0495 (13)	0.090 (2)	0.0631 (17)	0.0111 (13)	0.0178 (12)	0.0067 (15)
C25	0.0396 (13)	0.094 (2)	0.108 (3)	−0.0015 (13)	0.0194 (15)	0.0143 (19)

### Di-µ-chlorido-1:4κ2Cl:Cl-2:3κ2Cl:Cl-dichlorido-2κCl,4κCl-bis[µ3-ethoxy(pyridin-2-yl)methanolato-1:2:3κ3O:N,O:O;1:3:4κ3O:O:N,O]bis[µ2-ethoxy(pyridin-2-yl)methanolato-1:2κ3N,O:O;3:4κ3N,O:O]tetracopper(II) (II) Geometric parameters (Å, º)

**Table:**

Cu1—O15	1.9170 (13)	C17—C18	1.369 (4)
Cu1—O26	1.9684 (13)	C17—H17	0.9300
Cu1—N10	1.9886 (17)	C20—C19	1.383 (4)
Cu1—Cl3	2.2181 (6)	C20—H20	0.9300
Cu1—O26^i^	2.4280 (14)	C13—C14	1.484 (3)
Cu1—Cu2	3.0122 (4)	C13—H13A	0.9700
Cu2—O15	1.9324 (13)	C13—H13B	0.9700
Cu2—O26	1.9707 (14)	C18—C19	1.370 (5)
Cu2—N21	1.9827 (17)	C18—H18	0.9300
Cu2—Cl4	2.1987 (7)	C9—C8	1.371 (3)
O15—C11	1.370 (2)	C9—H9	0.9300
O26—C22	1.386 (2)	C6—C7	1.385 (4)
O12—C11	1.409 (2)	C6—H6	0.9300
O12—C13	1.426 (3)	C19—H19	0.9300
O23—C24	1.413 (3)	C24—C25	1.494 (4)
O23—C22	1.418 (2)	C24—H24A	0.9700
N21—C16	1.339 (3)	C24—H24B	0.9700
N21—C20	1.341 (3)	C7—C8	1.365 (4)
N10—C5	1.333 (3)	C7—H7	0.9300
N10—C9	1.336 (3)	C8—H8	0.9300
C5—C6	1.378 (3)	C14—H14A	0.9600
C5—C11	1.509 (3)	C14—H14B	0.9600
C11—H11	0.9800	C14—H14C	0.9600
C16—C17	1.384 (3)	C25—H25A	0.9600
C16—C22	1.506 (3)	C25—H25B	0.9600
C22—H22	0.9800	C25—H25C	0.9600
			
O15—Cu1—O26	78.21 (6)	O26—C22—O23	109.23 (15)
O15—Cu1—N10	81.81 (6)	O26—C22—C16	106.85 (16)
O26—Cu1—N10	156.02 (7)	O23—C22—C16	107.52 (17)
O15—Cu1—Cl3	176.95 (5)	O26—C22—H22	111.0
O26—Cu1—Cl3	100.34 (4)	O23—C22—H22	111.0
N10—Cu1—Cl3	98.95 (5)	C16—C22—H22	111.0
O15—Cu1—O26^i^	92.55 (5)	C18—C17—C16	118.3 (3)
O26—Cu1—O26^i^	79.23 (6)	C18—C17—H17	120.9
N10—Cu1—O26^i^	115.02 (6)	C16—C17—H17	120.9
Cl3—Cu1—O26^i^	89.80 (4)	N21—C20—C19	120.3 (3)
O15—Cu1—Cu2	38.69 (4)	N21—C20—H20	119.8
O26—Cu1—Cu2	40.15 (4)	C19—C20—H20	119.8
N10—Cu1—Cu2	117.54 (5)	O12—C13—C14	108.1 (2)
Cl3—Cu1—Cu2	139.425 (18)	O12—C13—H13A	110.1
O26^i^—Cu1—Cu2	90.19 (3)	C14—C13—H13A	110.1
O15—Cu2—O26	77.80 (6)	O12—C13—H13B	110.1
O15—Cu2—N21	157.61 (7)	C14—C13—H13B	110.1
O26—Cu2—N21	80.81 (7)	H13A—C13—H13B	108.4
O15—Cu2—Cl4	100.71 (5)	C17—C18—C19	119.8 (2)
O26—Cu2—Cl4	170.05 (5)	C17—C18—H18	120.1
N21—Cu2—Cl4	99.21 (6)	C19—C18—H18	120.1
O15—Cu2—Cu1	38.33 (4)	N10—C9—C8	122.3 (2)
O26—Cu2—Cu1	40.09 (4)	N10—C9—H9	118.9
N21—Cu2—Cu1	119.47 (6)	C8—C9—H9	118.9
Cl4—Cu2—Cu1	136.30 (2)	C5—C6—C7	118.5 (2)
C11—O15—Cu1	118.00 (12)	C5—C6—H6	120.8
C11—O15—Cu2	136.01 (13)	C7—C6—H6	120.8
Cu1—O15—Cu2	102.98 (6)	C18—C19—C20	119.8 (3)
C22—O26—Cu1	127.03 (12)	C18—C19—H19	120.1
C22—O26—Cu2	111.56 (12)	C20—C19—H19	120.1
Cu1—O26—Cu2	99.76 (6)	O23—C24—C25	109.2 (2)
C22—O26—Cu1^i^	113.05 (11)	O23—C24—H24A	109.8
Cu1—O26—Cu1^i^	100.77 (6)	C25—C24—H24A	109.8
Cu2—O26—Cu1^i^	101.06 (5)	O23—C24—H24B	109.8
C11—O12—C13	113.30 (16)	C25—C24—H24B	109.8
C24—O23—C22	114.72 (18)	H24A—C24—H24B	108.3
C16—N21—C20	119.9 (2)	C8—C7—C6	119.3 (2)
C16—N21—Cu2	113.18 (14)	C8—C7—H7	120.4
C20—N21—Cu2	126.56 (17)	C6—C7—H7	120.4
C5—N10—C9	118.78 (19)	C7—C8—C9	119.1 (2)
C5—N10—Cu1	113.66 (13)	C7—C8—H8	120.5
C9—N10—Cu1	126.96 (16)	C9—C8—H8	120.5
N10—C5—C6	122.1 (2)	C13—C14—H14A	109.5
N10—C5—C11	115.44 (17)	C13—C14—H14B	109.5
C6—C5—C11	122.4 (2)	H14A—C14—H14B	109.5
O15—C11—O12	113.60 (16)	C13—C14—H14C	109.5
O15—C11—C5	109.41 (16)	H14A—C14—H14C	109.5
O12—C11—C5	103.56 (16)	H14B—C14—H14C	109.5
O15—C11—H11	110.0	C24—C25—H25A	109.5
O12—C11—H11	110.0	C24—C25—H25B	109.5
C5—C11—H11	110.0	H25A—C25—H25B	109.5
N21—C16—C17	121.9 (2)	C24—C25—H25C	109.5
N21—C16—C22	114.57 (17)	H25A—C25—H25C	109.5
C17—C16—C22	123.6 (2)	H25B—C25—H25C	109.5

Symmetry code: (i) −*x*+1, −*y*+1, −*z*+1.

### Di-µ-chlorido-1:4κ2Cl:Cl-2:3κ2Cl:Cl-dichlorido-2κCl,4κCl-bis[µ3-ethoxy(pyridin-2-yl)methanolato-1:2:3κ3O:N,O:O;1:3:4κ3O:O:N,O]bis[µ2-ethoxy(pyridin-2-yl)methanolato-1:2κ3N,O:O;3:4κ3N,O:O]tetracopper(II) (II) Hydrogen-bond geometry (Å, º)

**Table:**

*D*—H···*A*	*D*—H	H···*A*	*D*···*A*	*D*—H···*A*
C9—H9···Cl3	0.93	2.78	3.333 (3)	119
C20—H20···Cl4	0.93	2.90	3.393 (3)	115
C13—H13*B*···Cl3^i^	0.97	2.82	3.787 (3)	173
C22—H22···O12^i^	0.98	2.66	3.578 (3)	156
C18—H18···O23^ii^	0.93	2.44	3.367 (3)	177

Symmetry codes: (i) −*x*+1, −*y*+1, −*z*+1; (ii) −*x*+3/2, *y*−1/2, −*z*+3/2.
